# Generating social network data using partially described networks: an example informing avian influenza control in the British poultry industry

**DOI:** 10.1186/1746-6148-7-66

**Published:** 2011-10-25

**Authors:** Sema Nickbakhsh, Louise Matthews, Paul R Bessell, Stuart WJ Reid, Rowland R Kao

**Affiliations:** 1 Boyd Orr Centre for Population and Ecosystem Health, Institute for Biodiversity, Animal Health and Comparative Medicine, University of Glasgow, Bearsden Road, Scotland, G61 1QH, UK; 2 Current address: Royal Veterinary College, University of London, Hawkshead Lane, North Mymms, Hatfield, Hertfordshire, AL9 7TA, UK

## Abstract

**Background:**

Targeted sampling can capture the characteristics of more vulnerable sectors of a population, but may bias the picture of population level disease risk. When sampling network data, an incomplete description of the population may arise leading to biased estimates of between-host connectivity. Avian influenza (AI) control planning in Great Britain (GB) provides one example where network data for the poultry industry (the Poultry Network Database or PND), targeted large premises and is consequently demographically biased. Exposing the effect of such biases on the geographical distribution of network properties could help target future poultry network data collection exercises. These data will be important for informing the control of potential future disease outbreaks.

**Results:**

The PND was used to compute between-farm association frequencies, assuming that farms sharing the same slaughterhouse or catching company, or through integration, are potentially epidemiologically linked. The fitted statistical models were extrapolated to the Great Britain Poultry Register (GBPR); this dataset is more representative of the poultry industry but lacks network information. This comparison showed how systematic biases in the demographic characterisation of a network, resulting from targeted sampling procedures, can bias the derived picture of between-host connectivity within the network.

**Conclusions:**

With particular reference to the predictive modeling of AI in GB, we find significantly different connectivity patterns across GB when network estimates incorporate the more demographically representative information provided by the GBPR; this has not been accounted for by previous epidemiological analyses. We recommend ranking geographical regions, based on relative confidence in extrapolated estimates, for prioritising further data collection. Evaluating whether and how the between-farm association frequencies impact on the risk of between-farm transmission will be the focus of future work.

## Background

Targeted collation of contact data typically only represent a small subset of the true population, and if these data are biased this may lead to misinterpretation of recorded contact structures [1-3]. Consequently, heterogeneities in population contact structure can be poorly characterised. The importance of such contact heterogeneities for infectious disease transmission have been highlighted through the development of social network models in humans [4] and movement network models in livestock [5-10]. In Great Britain (GB), the application of network analysis to livestock movements has been uniquely informed by a well-defined temporally explicit Cattle Tracing System (CTS) database [11,12]. However, even in this case there is some evidence of potential bias in cattle movement patterns arising through missing or incorrect movement records at the level of the type of enterprise [13]. Such systematic errors, arising from data collection procedures and inaccuracies in reported information, may lead to biases in the quantification of network properties. Bias identification is therefore an important step in ensuring model validity.

Mathematical models of avian influenza (AI) in Great Britain (GB) have been largely informed by the Poultry Network Database (PND), providing poultry network information for a subset of the industry, and the Great Britain Poultry Register (GBPR) which provides more representative demographic information. Although the PND does not reflect temporally explicit movements on-to and off-of farms, shared industry associations have been used to infer potential contacts between farms and have informed stochastic simulation and exploratory models [14-16]. For example, all farms that are associated with a particular slaughterhouse are assumed potentially epidemiologically linked to one another. In the absence of epidemic data, and therefore without the ability to validate predictive models for AI control in GB, mathematical models are a valuable tool for exploring the connectivity of the poultry industry. These epidemiological models have investigated the efficacy of current control measures for AI in GB and have identified particular scenarios that could result in a large outbreak [14-16].

The PND was collated in 2006 by the Veterinary Laboratories Agency (VLA). This was designed to establish farms that share industry associations such as through catching companies (CCs), slaughterhouses (SHs) or through being part of a larger integrated company (IC). From this, an estimate of between-farm association frequency (i.e. the maximum number of farms a single farm may be associated with) can be made at a farm-level, which can be used to inform logistical considerations during a disease outbreak prior to the implementation of movement restrictions [17]. These between-farm associations inferred from the PND have been used as a proxy for between-farm "contacts" as they are considered to represent potential routes of between-farm spread of infection through personnel, shared equipment and vehicles [16].

Epidemiological evidence from previous outbreaks of AI indicate the role of indirect transmission via fomites, for example through shared equipment, the reuse of disposable egg-trays, the movement of vehicles (during chick delivery, the delivery of feed, and the collection of dead-birds), the management practices of integrated companies, contaminated bird-carrying crates during slaughterhouse-related farm visits and through the clothing, shoes and hands of farm visitors [18-27]. Such mechanisms of transmission via fomites are also identified as sources of possible risk through catching company personnel and vehicles associated with slaughterhouse-related farm visits [28].

Whilst this evidence is largely circumstantial, arising from epidemiological investigations, it is considered likely that AI will share the same mechanisms for between-farm transmission as other pathogens similarly transmitted via the faecal-oral route [29], such as *Salmonella*, *Campylobacter *and those associated with coccidiosis [16]. Fomites have been implicated in poultry flock infections caused by these pathogens and represent possible mechanisms of between-farm transmission; for example, during slaughterhouse-related farm visits via equipment such as bird-carrying crates and pallets, the wheels of forklift trucks and slaughterhouse vehicles, the boots of drivers' and catchers', as well as via staff and equipment shared between different farm premises [20,30-34]. Evidence from previous outbreaks also suggests that spatial spread, possibly via airborne mechanisms, may also play an important role between farms within close proximity [18,20,25,35,36]. However, this mechanism is considered to be relatively less important for GB compared with countries such as the Netherlands [35], which has regions of greater poultry farm density.

As a result of the targeted sampling of known SHs and CCs, missing data inherently biases the PND towards large poultry premises. Therefore the PND cannot be considered representative of the entire GB poultry industry and was never intended to be so [Lucy Snow, pers. comm.]. It has been shown that even when individuals are sampled at random, this process may not result in a random representation of their contacts, and consequently overall network properties [1,2,37]. Missing data within the PND are inherently non-random, and therefore systematic differences in the types of farms sampled compared to those unsampled may further exacerbate the misrepresentation of network properties, and the identification of high risk sectors of the poultry industry. The validity of generalising PND informed network properties to a national-scale is potentially reduced by missing farms. Therefore, establishing the likely characteristics of these missing farms, based on the known properties of those that are well-characterised, is an important step to inform future data collection exercises. It is only through a more representative characterisation of the poultry industry that contact heterogeneities can be usefully applied to predictive models of poultry disease control.

To our knowledge, the appropriateness of using inferred industry contacts from the PND for informing predictive AI models in GB has not been considered in the published literature. In particular, the potential implications of targeted sampling procedures for predictive modelling of AI control have yet to be quantified. Potential biases in inferred poultry network properties may have important consequences for government preparedness of resource distribution during an outbreak; the extent of between-farm spread may depend on how rapid and where the movement restrictions that inhibit this risk are implemented. As the human health, animal welfare and economic consequences of a large AI outbreak could potentially be catastrophic [38-44], government and industry preparedness for such an event is vital.

Our aim was to identify geographical areas with biases in the farm contact structure by extrapolating network data informed by the PND to the GBPR, which is more demographically representative of GB poultry farms but without the detailed information on between-farm associations via the poultry industry. This database was established by the British Department for Environment, Food and Rural Affairs (Defra) in December 2005, and it is mandatory for all commercial farms holding more than 50 birds to record their farm-related details [45].

Specifically, our objectives were to: (i) determine statistical associations between farm-level factors and network informed between-farm association frequency, using multivariable logistic regression; (ii) extrapolate the fitted statistical models to each farm recorded in the GBPR, obtaining predicted probabilities for categorical between-farm association frequency; (iii) compare the regional-level (GB divided into eleven geographical regions) distribution of PND-informed between-farm association frequencies with estimates following extrapolation to the GBPR.

## Results

### The poultry industry network

**Figure 1:**
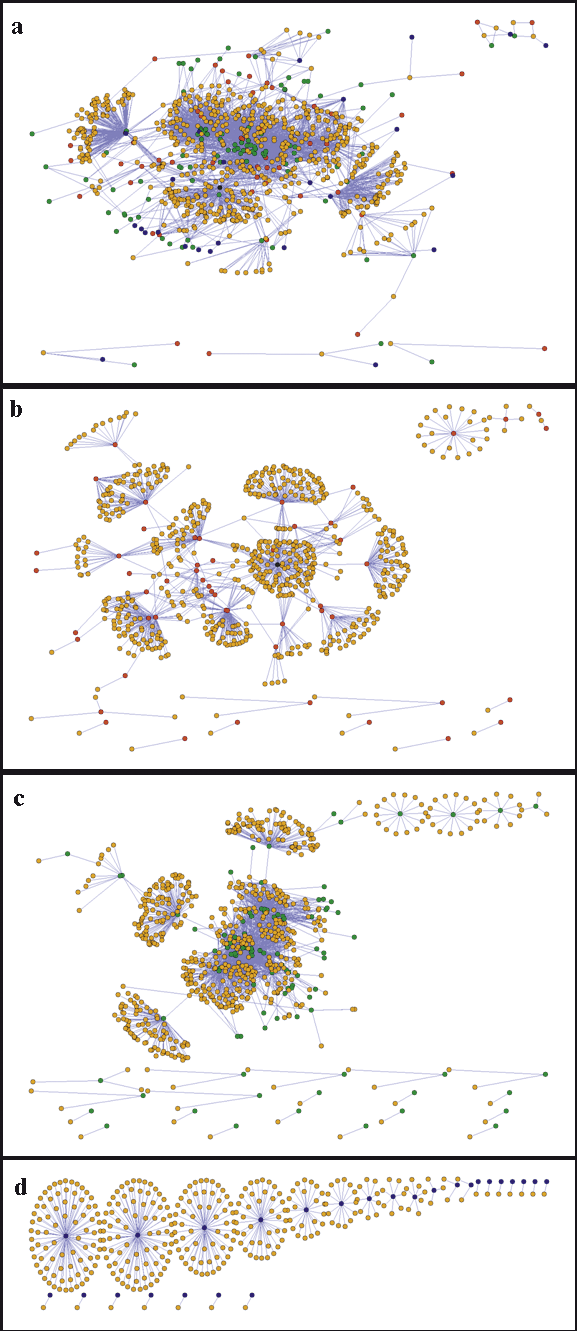
**Components of the British poultry industry network**. Full contact network between poultry farms, slaughterhouses (SHs), catching companies (CCs) and integrated companies (ICs) (a), and network components partitioned into associations between farms and SHs (b), farms and CCs (c) and between farms within ICs (d), using farms for which complete contact information was known (n = 662). Orange = farm, red = SH, black = largest SH, green = CC, blue = IC.

The PND, with between-farm associations assumed to arise through shared industry contacts, was highly connected: most farms were potentially associated with almost all other farms, mostly through slaughterhouses (SHs) and catching companies (CCs) (Figures 1). This is consistent with previous work using the PND which reports that, when all types of industry contacts are combined, the giant component of the network (i.e. the largest group of connected farms) includes the majority of premises [16]. The largest SH is important for connecting smaller clusters of farms that are themselves connected to each other through SHs (Figure 1b).

### Assessing the introduction of bias following data reduction

The univariable odds ratios (ORs), computed both before and after the exclusion of farm records with missing predictor variable data (see Methods section), did not suggest that any significant biases would be introduced to either the scenario 1 or 2 analyses (Tables 1 and 2 respectively). Therefore the reduced dataset was used for the multivariable statistical modelling.

**Table 1:** Crude odds ratios before and after removal of records with missing data: scenario 1 analyses

	Full data (n = 662 farms)			Following the removal of records (n = 348 farms)			
**Farm-level predictors**	**OR^a^**	**s.e. OR^b^**	**p-value**	**OR^a^**	**s.e. OR^b^**	**p-value**	**% change**

L vs. S bird count^e^	0.284	1.292	<0.0001	0.185	1.367	<0.0001	34.9^c^
L vs. S house count^e^	0.354	1.306	<0.0001	0.299	1.327	<0.0001	15.5
Indoors	0.298	1.289	<0.0001	0.301	1.307	<0.0001	1.0
Free-range	5.266	1.298	<0.0001	5.010	1.317	<0.0001	4.9
Housing other	0.829	1.510	0.650	0.598	1.590	0.268	27.9^c^
Partial housing	1.009	1.941	0.990	0.763	2.209	0.733	24.4
Integrated	0.271	1.222	<0.0001	0.258	1.309	<0.0001	4.8
East	1.905	1.298	0.014	2.545	1.392	0.005	25.1
Scotland	0.333	2.144	0.150	0.308	2.899	0.268	7.5
Wales	0.119	2.087	0.004	0.192	2.848	0.115	38.0^d^
West	0.418	1.287	0.001	0.401	1.429	0.010	4.1

^a^OR = odds ratio; ^b^s.e. = standard error of the odds ratio; ^c ^>25% change in odds ratio but direction of association and significance is comparable; ^d^single variable for which there is >25% change in odds ratio and no change in direction of association, but significance is altered; ^e^L = large, S = small.

**Table 2:** Crude odds ratios before and after removal of records with missing data: scenario 2 analyses

	Full data (n = 662 farms)			Following the removal of records (n = 348 farms)			
**Farm-level predictors**	**OR^a^**	**s.e. OR^b^**	**p-value**	**OR^a^**	**s.e. OR^b^**	**p-value**	**% change**

L vs. S bird count^d^	7.304	1.485	<0.0001	5.588	1.369	<0.0001	23.5
L vs. S house count^d^	1.783	1.289	0.023	1.879	1.310	0.019	5.1
Indoors	2.888	1.425	0.003	3.822	1.472	0.001	24.4
Free-range	0.280	1.529	0.003	0.227	1.579	0.001	18.9
Housing other	0.363	1.648	0.043	0.338	1.656	0.031	6.9
Partial housing	0.890	1.991	0.866	0.592	2.254	0.518	33.5^c^
Integrated	0.544	1.232	0.003	0.554	1.319	0.033	1.8
East	0.446	1.401	0.017	0.426	1.538	0.047	4.5
Scotland	0.061	2.837	0.007	0.090	2.914	0.024	32.2^c^
Wales	0.090	1.722	<0.0001	0.116	2.176	0.006	22.4
West	0.047	1.477	<0.0001	0.056	1.583	<0.0001	16.1

^a^OR = odds ratio; ^b^s.e. = standard error of the odds ratio; ^c ^>25% change in odds ratio but direction of association and significance is comparable; ^d^L = large, S = small.

### Scenario 1: predictors of large between-farm association frequency

Equation 1 shows the form of the logistic model used to identify predictors of a large between-farm association frequency (L_af_; referred to as scenario 1, see Methods for further details). The logit function represents a nonlinear transformation of the probability that farm *i *has a L_af_, Pr(L_af,*i*_), *β_0_*is the average log-odds of a L_af _for farms within the baseline predictor variable categories, *β_1, _β_2 _... β_11 _*are average log-ORs for each predictor variable (see Tables 3 and 4 for definitions of the linear predictors), *β_12 _β_13 _*and *β_14 _*are the log-ORs for farms in the baseline categories for interacting variables.

**Table 3:** Farm-level predictors used in statistical analyses of associations with between-farm association frequency

Predictor variable	Data type	Description	% missing (n = 662)
Species	Categorical	Production type or poultry species	54.38
Bird count ^a^	Binary (large vs. small)	Total number of birds on site	41.39
House count ^a^	Binary (large vs. small)	Total number of poultry houses on site	40.03
Indoors ^b^	Binary (yes vs. no)	Categorisation of whether the premises houses any of its birds indoors (e.g. barn, cage or pole barn)	38.97
Outdoors ^b^	Binary (yes vs. no)	Categorisation of whether the premises houses any of its birds outside	38.97
Free-range ^b^	Binary (yes vs. no)	Categorisation of whether the premises has registered any free range birds	38.97
Housing other ^b^	Binary (yes vs. no)	Categorisation of whether the premises keeps any of its birds in other housing	38.97
Partial housing ^b^	Binary (yes vs. no)	Categorisation of whether the premises keeps any of its birds in partial housing (e.g. coop, brooder house, shelter pen or grass run)	38.97
Integrated	Binary (yes vs. no)	Whether premises is part of an integrated company or associated with a company	6.95
Region	Categorical (East^c^, Scotland, Wales, West^c ^vs. North^c^)	Regional location of premises within GB based on the county in the premises address	8.61

^a^Following categorisation of original numeric variables

^b ^Original categorisation for variables indicating the farm management type

^c^Geographical regions of England

**Table 4:** Definitions of farm-level predictors grouped into their cross classifications as used in statistical analyses

Original variables*	Description of cross-classification	Predictor variable ID	Farm frequency
House count/ Bird count	house count = small, bird count = small	hbSS	128
	house count = small, bird count = large	hbSL	53
	house count = large, bird count = small	hbLS	50
	house count = large, bird count = large	hbLL	117
Indoor/ Free-range	indoor = no, free-range = no	ifNN	26
	indoor = no, free-range = yes	ifNY	79
	indoor = yes, free-range = no	ifYN	229
	Indoor = yes, free-range = yes	ifYY	14

*see Table 3

(1) $$\begin{array}{c} \text{logit Pr(}{\text{L}}_{\text{af,}i}\text{) }=\beta_{0}+\beta_{1}hbLS_{i}+\beta_{2}hbSL_{i}\\ +\beta_{3}hbLL_{i}+\beta_{4}ifNN_{i}+\beta_{5}ifNY_{i}+\beta_{6}ifYY_{i}\\ +\beta_{7}Integrated_{i}+\beta_{8}East_{i}+\beta_{9}Scotland_{i}\\ +\beta_{10}Wales_{i}+\beta_{11}West_{i}+\beta_{12}ifNN_{i}*Integrated_{i}\\ +\beta_{13}ifNY_{i}*Integrated_{i}+\beta_{14}ifYY_{i}*Integrated_{i} \end{array}$$

Management type and poultry house count were found to be significantly associated with between-farm association frequency (Table 5); farms keeping only free-range birds were more likely (OR = 12.19, 95% CI = 3.82-38.91, p < 0.001), and farms with a large poultry house count were less likely (OR = 0.16, 95% CI = 0.04-0.64, p = 0.009 and OR = 0.32, 95% CI = 0.14-0.71, p = 0.005, for farms with small and large bird counts respectively) to be assigned L_af. _There was also evidence of association with geographical location; farms located in the West of England were less likely than farms located in the North of England to be assigned L_af _(OR = 0.32, 95% CI = 0.14-0.76, p = 0.01). The effect of management type was found to differ depending on the integration status of the farm; free-range integrated farms were significantly less likely than free-range non-integrated farms to be assigned L_af _and vice versa (interaction coefficient = 0.13, 95% CI = 0.03-0.59, p = 0.009). There was no evidence of a poor fit to the data based on an assessment of the model residuals or model predictive ability (area under the ROC curve for varying model sensitivity and specificity = 0.86).

**Table 5:** Results from multivariable logistic regression: scenario 1 analyses (n = 348 farms)

Farm-level predictor	Predictor levels^a^	OR^b^	lower 95% CI^c^	upper 95% CI^c^	p-value
Intercept^d^	-	0.608	0.264	1.401	0.243

House count/Bird count	hbSS^e^	1	-	-	-
	**hbLS**	**0.161**	**0.041**	**0.636**	**0.009**
	hbSL	0.507	0.207	1.246	0.139
	**hbLL**	**0.317**	**0.141**	**0.711**	**0.005**

Indoor/Free-range	ifYN^e^	1	-	-	-
	ifNN	1.810	0.275	11.927	0.537
	**ifNY**	**12.185**	**3.815**	**38.913**	**<0.001**
	ifYY	3.072	0.725	13.018	0.128

Integration status	Non-integrated^e^	1			
	Integrated	0.681	0.307	1.512	0.345

Geographical location	Region: North^e^	1	-	-	-
	Region: East	1.337	0.567	3.152	0.507
	Region: Scotland	0.324	0.038	2.752	0.302
	Region: Wales	0.126	0.012	1.329	0.085
	**Region: West**	**0.321**	**0.135**	**0.761**	**0.010**

Interaction terms	ifNN*Integrated	0.900^f^	0.075	10.778	0.934
	ifNY* Integrated	**0.128^f^**	**0.028**	**0.594**	**0.009**
	ifYY* Integrated	0.965^f^	0.048	19.526	0.982

^a^See tables 3 and 4 for definitions; ^b^OR = odds ratio; ^c^CI = confidence interval of the OR; ^d^average odds in the baseline predictor groups; ^e^reference-level category; ^f^OR for farms in the baseline category of the other interacting variable; results significant at 5% error level are indicated in bold.

### Scenario 2: predictors of medium between-farm association frequency

Equation 2 shows the form of the logistic model used to identify predictors of a medium between-farm association frequency (M_af_; referred to as scenario 2, see Methods for further details). The logit function represents a nonlinear transformation of the probability that farm *i *has a M_af_, Pr(M_af,*i*_), *β_0_*is the average log-odds of a M_af _for farms within the baseline predictor variable categories and β_*1*_, β_*2 *_... β_8 _are average log-ORs for each predictor variable (see Tables 3 and 4 in the methods for definitions of the linear predictors).

(2) $$\begin{array}{c} \text{logit Pr(}{\text{M}}_{\text{af,}i}\text{) }=\beta_{0}+\beta_{1}hbLS_{i}+\beta_{2}hbSL_{i}\\ +\beta_{3}hbLL_{i}+\beta_{4}Integrated_{i}+\beta_{5}East_{i}\\ +\beta_{6}Scotland_{i}+\beta_{7}Wales_{i}+\beta_{8}West_{i} \end{array}$$

In contrast to scenario 1 analyses, bird count rather than poultry house count was a significant predictor of between-farm association frequency (Table 6). Farms with a large bird count were significantly more likely to be assigned M_af _(OR = 6.89, 95% CI = 2.18-21.76, p = 0.001 and OR = 6.22, 95% CI = 2.25-17.25, p < 0.001, for farms with small and large poultry house counts respectively). Similarly to scenario 1 analyses, integrated companies were significantly less likely than non-integrated companies to be assigned M_af _(OR = 0.44, 95% CI = 0.21-0.92, p = 0.03). Geographic location was also found to be important; farms located in Scotland, Wales and the West of England were significantly less likely than farms located in the North of England to be assigned M_af _(ORs = 0.045 to 0.073, p ≤ 0.005). There was no evidence of a poor fit to the data based on an assessment of the model residuals or model predictive ability (area under the ROC curve for varying model sensitivity and specificity = 0.83).

**Table 6:** Results from multivariable logistic regression: scenario 2 analyses (n = 270 farms)

Farm-level predictor	Predictor levels^a^	OR^b^	lower 95% CI^c^	upper 95% CI^c^	p-value
Intercept^d^	-	0.603^d^	0.234	1.553	0.294

House count/Bird count	hbSS^e^	1	-	-	-
	hbLS	0.148	0.016	1.352	0.091
	**hbSL**	**6.891**	**2.182**	**21.762**	**0.001**
	**hbLL**	**6.224**	**2.246**	**17.247**	**<0.001**

Integration status	Non-integrated^e^	1	-	-	-
	**Integrated**	**0.442**	**0.212**	**0.922**	**0.030**

Geographical location	Region: North^e^	1	-	-	-
	Region: East	0.510	0.200	1.301	0.159
	**Region: Scotland**	**0.045**	**0.005**	**0.396**	**0.005**
	**Region: Wales**	**0.073**	**0.015**	**0.362**	**0.001**
	**Region: West**	**0.050**	**0.019**	**0.130**	**<0.001**

^a^See tables 3 and 4 for definitions; ^b^OR = odds ratio; ^c^CI = confidence interval of the OR; ^d^average odds in the baseline predictor groups; ^e^reference-level category; results significant at 5% error level are indicated in bold.

### Comparative analysis of geographical variation

**Figure 2:**
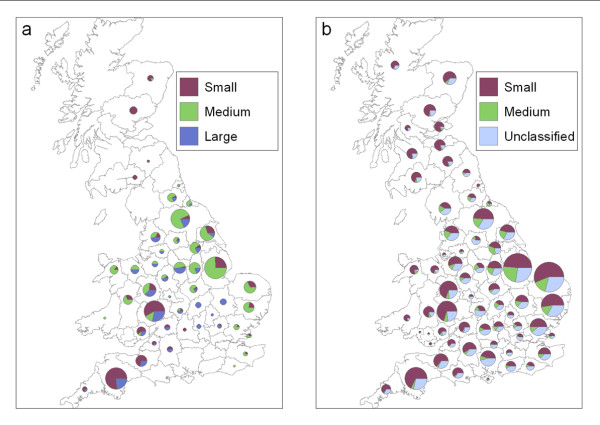
**Extrapolating between-farm association frequency from the Poultry Network Database to the Great Britain Poultry Register**. County-level average probabilities of small, medium and large between-farm association frequencies, as observed in the Poultry Network Database (n = 662) (a), and as predicted following extrapolation to the Great Britain Poultry Register (GBPR) (n = 3009) using fitted statistical models (farms known to be associated with the large slaughterhouse represent only ~3% of GBPR farms and therefore cannot be seen from this figure) (b). Pie sizes are proportional to the county-level number of farms for the respective datasets.

**Figure 3:**
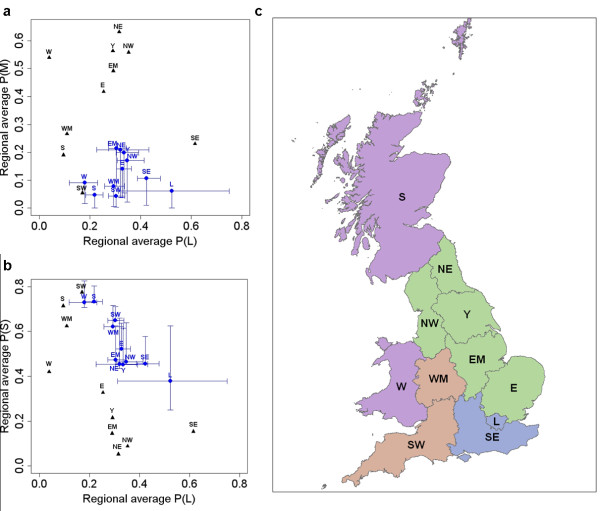
**Predicted regional-level between-farm association frequency extrapolated to farms recorded in the Great Britain Poultry Register**. Regional average probabilities of (a) large versus medium and (b) large versus small between-farm association frequencies (blue circles), following extrapolation of network information to the Great Britain Poultry Register (n = 3009 farms). Error bars represent 95% confidence intervals generated from 1000 stochastic simulations of randomly assigning each farm to a small, medium or large between-farm association frequency group. Black triangles represent proportions of farms within these categories observed from the Poultry Network Database (n = 662 farms). (c) Geographical clustering of the regional predicted probabilities represented by their corresponding colours (note: Scotland and Wales were considered distinct from the other regions). W = Wales; S = Scotland; L = Greater London; WM = West Midlands, SW = South West, EM = East Midlands, NE = North East, E = East, Y = Yorkshire, NW = North West and SE = South East of England.

Comparing the PND with the GBPR, the geographical distribution of sampling coverage and capacity was noticeably different (Figures 2a and 2b). It is possible that this misrepresentation of farms within the PND has lead to systematic error (or bias) in the inherent description of the network. Indeed, following the extrapolation of between-farm association frequency to the GBPR, substantial differences were found when compared to the observations from the PND. Comparing both datasets, the probabilities obtained were significantly different for all regions (Figures 3a and 3b); the values inferred from the PND do not overlap the 95% confidence intervals (CIs) generated for the estimates obtained using the GBPR data (see Methods section for further details on the simulations used to generate these CIs).

Comparing the regions within Great Britain, geographical variation in the predicted probabilities extrapolated to the GBPR data was observed; neighbouring regions were found to be typically more similar to each other. For example, three regional clusters were observed: (i) the North West, North East, Yorkshire, East Midlands and Eastern regions of England, (ii) Greater London and the South East of England, and (iii) the West Midlands and South West of England (Figure 3c). Scotland and Wales on the other hand appear distinct; their large between-farm association frequency propensity is different to the other regions (i.e. the 95% CIs do not overlap the other regions), whilst they appear more similar in terms of their medium between-farm association frequency probabilities (Figures 3a and 3b). Furthermore, the width of the CIs generated using the GBPR demonstrates our confidence in these estimates and whether their likely range is comparable between regions. Prioritising regions based on the rank order of our confidence in the estimated probabilities (i.e. more confidence can be ascribed to a narrower CI) reveals differences across the between-farm association frequency categories (Table 7).

**Table 7:** British regions ranked by confidence interval widths for estimated probabilities of between-farm association frequencies

Regions ranked by L ^a^	CI range for L ^b^	Regions ranked by M ^a^	CI range for M ^b^	Regions ranked by S ^a^	CI range for S ^b^
Greater London	0.438	North East	0.132	East	0.065
North East	0.208	Wales	0.056	South West	0.069
North West	0.106	North West	0.048	West Midlands	0.074
Wales	0.103	East Midlands	0.044	Scotland	0.075
South East	0.092	Yorkshire	0.043	East Midlands	0.076
Yorkshire	0.089	South East	0.032	South East	0.092
West Midlands	0.074	East	0.027	Yorkshire	0.096
Scotland	0.072	West Midlands	0.023	North West	0.117
South West	0.069	Scotland	0.016	Wales	0.119
East Midlands	0.068	South West	0.015	North East	0.226
East	0.060	Greater London	<0.001	Greater London	0.375

^a^Regions ranked in order of priority based on confidence in predicted probabilities of large (L) or medium (M) between-farm association frequency (CI range ranked from highest to lowest), and small (S) between-farm association frequency (CI range ranked from lowest to highest).

^b^The 95% confidence interval range (upper bound - lower bound) for predicted probabilities of large (L), medium (M), and small (S), between-farm association frequencies.

## Discussion

### Geographical bias in network data

The targeted sampling strategies employed in the collation of network data for epidemiological use may be inherently biased in terms of demographic representation. Our results demonstrate how such demographic information may also result in a biased representation of the network properties. Using an example of the British poultry industry network comprised of farms, slaughterhouses (SHs), catching companies (CCs) and integrated companies (ICs), we show how risk-based collation of the PND has potentially led to misrepresentation of between-farm connectivity. These findings also have importance for other poultry diseases also transmitted via fomites, such as *Salmonella*, *Campylobacter *and those associated with coccidiosis [31-33,46,47]. Our results have particular implications for highly pathogenic AI (HPAI) in GB, as predictive and exploratory models have been informed by the network structure provided by the PND [14-16].

Although the PND was considered *a priori *to be inherently biased in terms of its representation of farm characteristics, bias in the network characteristics had not previously been explored. Our results show how the geographical distribution of between-farm association frequency, as inferred from the PND, significantly differed following extrapolation of this network data to the GBPR (Figures 3a and 3b). The purpose of this extrapolation process was not to accurately predict farm-level connectivity for farms recorded in the GBPR, and assumes the statistical association between the farm-level predictors and between-farm association frequency is true. Extrapolating this network information was a method by which to test the PND network associations making use of the more representative distribution of farm-level factors provided by the GBPR.

Our analyses have demonstrated heterogeneities in the demographic profile between the datasets, highlighting types of farms and regions of GB where network data should be expanded. The confidence intervals for probabilities of between-farm association frequencies, estimated for the GBPR data, reflect the accuracy of these estimates (Figures 3a and 3b). We recommend further sampling should be carried out within regions where we have relatively poor confidence in our estimates, in particular prioritising regions for which we have the smallest confidence in large between-farm association frequency probabilities (i.e. first column of Table 7).

### Methodological considerations

Using multivariable logistic regression we have identified statistically significant (p < 0.01) associations between farm-level factors and between-farm association frequency using the PND. We found that small (based on both the number of poultry houses and total bird count), non-integrated, free-range farms were more likely to have a large between-farm association frequency. Although our aim here was not to directly determine the impact of network biases on disease transmission predictions, drawing valid conclusions from analyses of contact heterogeneity requires consideration of systematic errors in sampled network data. The analyses here did not directly allow for such inference as between-farm association frequencies do not necessarily correlate with AI exposure frequencies. For example, although we found that free-range farms may have a greater overall between-farm association frequency, we would expect them to have fewer farm visits on a daily basis due to their typically longer production cycles and smaller bird throughput.

Nevertheless, the chance of a farm becoming exposed to AI virus during a slaughterhouse-related farm visit will depend in part on the number of farms visited by a single SH vehicle and catching team within a single day. We believe that it can be reasonably hypothesised that premises associated with larger SHs (i.e. with a greater number of associated farms), such as the free-range farms in our analyses, may have a greater risk of infection from other associated farms. This is because of the likely greater number of farm clients visited in one day by the vehicles of these larger SHs (up to a threshold level of a feasible number of daily farm visits) [Jennifer Dent, pers. comm.]. In the case of CC movements, an analysis of temporally explicit catching-related movement data suggests they may be relatively less important than SH vehicles for AI transmission, as only one farm was visited by a catching team within a single-day for 84% of the recorded farm visits; however, up to seven visits within a day was possible [48], and this result could be limited by the representation of only one CC.

One source of missing data within the PND results from non-reporting of information by at least one farmer across all data fields (Table 3). Although methods for imputing such missing values for the purpose of statistical regression analyses exist [49-51], such measures would likely add to the uncertainty in our extrapolated outputs and so were considered inappropriate for the purpose of the analysis here. In any case, it was determined unlikely that such non-reporting resulted in systematic errors in the estimated model coefficients, as no significant differences were identified from a comparison of univariate ORs calculated before and after the removal of records with missing data (Tables 1 and 2).

Existing analyses have used the PND without consideration to data biases. Truscott *et al. *(2007) used the PND to derive a negative binomial distribution for the number of contacts. Similarly, Sharkey *et al. *(2008) used the PND to inform the geographical profile and frequency of farm movements, and Dent *et al. *(2008) used the PND to infer farm associations through shared industry contacts (as in the analyses here). These studies have thereby potentially misrepresented the extent of network connectivity through the under-representation of smaller farms. Through better characterisation of these misrepresented sectors of the poultry industry, the use of poultry network data for informing predictive models of AI control can be more reliably assessed.

### Epidemiological implications

Our results suggest that free-range farms may have more extensive implications for AI control measures than previously anticipated. Free-range farms could be targeted both to minimise the risk of introduction through contact with wild birds, such as through targeted surveillance [52], and - via improved biosecurity measures - to minimise the risk of onward spread through SH vehicle movements. Furthermore, free-range farms may have comparatively different logistical considerations in terms of the extent of contact tracing due to their potential wide range of associations. These implications for disease control measures, to minimise between-farm spread via fomites during farm visits, are applicable to the period prior to the detection and notification of an outbreak to the authorities [17]. Once notification has occurred, the risk of between-farm spread will be limited to how rapid and where control measures are implemented, as well as to poultry farm density if airborne mechanisms of spread are important [35]. Whether the observed demographic bias in network connectivity does indeed correspond to infection risk will be the focus of future work incorporating temporally explicit CC movement data.

Using the PND to inform predictive models of AI control may also lead to a misrepresentation of maximum between-farm association frequency at a national-scale. The different implications for regional-level disease control between the datasets highlights the potential difficulties of relying upon data subsets to infer disease control at this scale. When comparing sampling coverage (the geographical distribution) and capacity (the proportion of the population captured) between the datasets alone, Scotland, the East and the South East of England appear particularly under-sampled by the PND (Figures 2a and 2b). However, significant under-estimation of large between-farm association frequency was found, when informed by the PND compared with the GBPR, for all regions except the South East and the North West of England (Figure 3). This suggests that the under-sampling of the PND is not alone predictive of bias in this network data.

We recommend that future data collection should target those farms where additional sampling could improve our confidence in estimated between-farm association frequencies. By ranking regions based on our confidence in these estimates we demonstrate how data collection can be prioritised, in particular in those regions where we have relatively low confidence in large between-farm association frequencies, such as Greater London and the North East of England (Table 7). We also highlight the apparent difference in large between-farm association frequency for Scotland and Wales, which appear distinct from the other regions despite their relatively narrow confidence intervals (Figure 3). Such differences between regions may be useful for informing targeted disease control strategies.

Future data collection should also be directed towards the subset of farms within the GBPR which were unclassified in terms of their probability of a large between-farm association frequency (see 'Extrapolating network data to the GBPR' in the Methods section). The farm-level predictors of large between-farm association frequency may only reflect the characteristics of farms connected to the large SH in the PND; it may not be appropriate to generalise and assume that farms with similar characteristics will also be associated with other large SHs. As the PND was deliberately targeted at larger poultry industry premises, the very large SH in the PND may represent the only one in GB of this size; however, the sampling procedure captured only 47.5% (57/120) of SHs approved by the British Food Standards Agency at the time these data were collated [Lucy Snow, pers. comm.]. Therefore, a better understanding of the activities of unsampled SHs is also important.

## Conclusions

We have shown how systematic errors in the demographic characterisation of network data, resulting from targeted sampling procedures, can bias the picture of between-host network connectivity. Detailed analyses of potential network bias within the PND are an important step towards obtaining a more accurate characterisation of the British poultry industry network structure. Providing a means of using this network information in a more representative way can help us more reliably infer the role of contact heterogeneities in the spread of poultry diseases. Based on the distribution of demographic factors represented by the GBPR, we have demonstrated that between-farm connectivity inferred from the PND may be biased. The sampling coverage and capacity is not alone indicative of this network bias; estimates of between-farm association frequency differed significantly across all regions of GB following extrapolation to the GBPR. We recommend that regions where we have relatively low confidence in our estimates of large between-farm association probability should be prioritised for future poultry network data collection. A subset of farms unsampled by the PND, and unclassified in terms of their large between-farm association frequency probability, were identified and we suggest these are also targeted in future data collection exercises. Evaluating whether and how the between-farm association frequencies impact on the risk of between-farm transmission will be the focus of future work.

## Methods

### Inferring between-farm association frequency

The PND consisted of surveys administered to: (i) single-site and (ii) multi-site farm premises, (iii) slaughterhouses (SHs) and (iv) catching companies (CCs), as informed by a NEEG (National Epidemiology Emergency Group) and CERA (Centre for Epidemiology and Risk Analysis) data collection exercise for Defra [53]. Catching companies comprise teams of personnel who are responsible for catching birds and loading them into vehicles for transportation to the SH. These companies may be independent and contracted by a SH, or employed by SHs or CCs who provide their own catching teams [28]. In total, these surveys provided information on 4,067 farms premises, 96 SHs and 102 CCs. These data were used to construct a between-farm association matrix, based on the assumption that farms that share the same SH, CC or through an integrated company (IC) were potentially epidemiologically linked, and therefore potential sources of AI virus exposure to each other [16].

SHs and CCs were considered to be independent industry layers, as CC teams and SH vehicles follow independent schedules, and so were considered to have different potential mechanisms of spreading AI between farms. For example, farms that share the same SH may share AI exposure indirectly through fomites via SH vehicles, should they visit multiple farms without disinfecting wheels or the bird carrying crates [32,54]. Farms that share the same CC may also share AI exposure risk through fomite transmission, but in this case via the wheels of vehicles transporting catching team personnel between-farms, forklift trucks, or through contamination of personnel clothing and equipment [19,33], and especially if they visit multiple farms within a single day [28]. The main risk to biosecurity results from the catchers footwear, clothing and masks/gloves if these are re-used on different poultry premises without sufficient disinfection [28]. A further potential contact mechanism was explored based on between-farm associations through ICs, to represent the risks associated with the movement of personnel and shared equipment by these farms [20,22]. No data were available for other potential mechanisms of transmission, such as through feed delivery [54,55], egg collection [26] or artificial insemination visits [56], and therefore are not represented here.

### Quantifying between-farm association frequency

**Figure 4:**
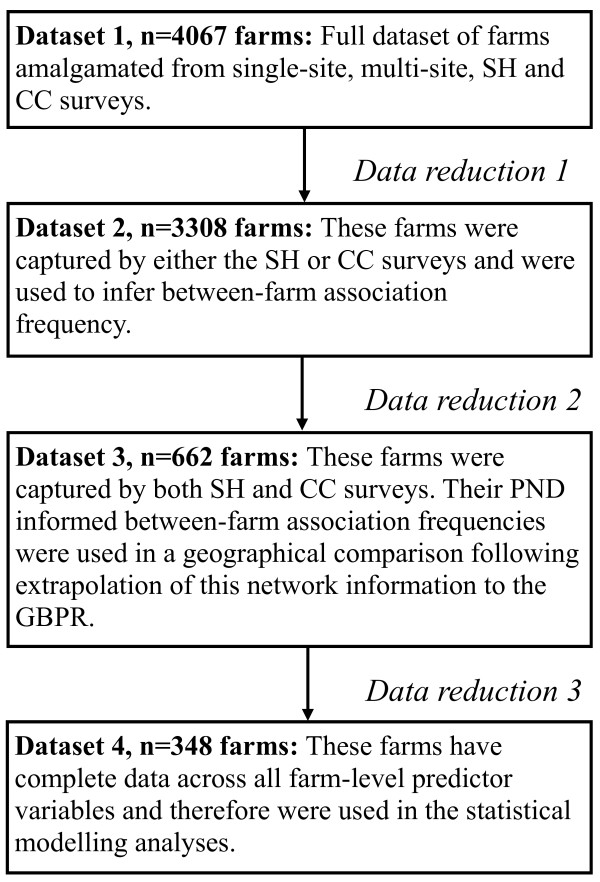
**Reducing the Poultry Network Database into data subsets**. SH = slaughterhouse; CC = catching company; PND = Poultry Network Database; GBPR = Great Britain Poultry Register.

A subset of farms captured by either the SH or CC surveys (n = 3308), and therefore for which only partial industry contact information was known, were used to inform the between-farm association matrix. This was considered appropriate as these farms contribute to the association-frequency of other farms captured by both surveys that were used in the statistical analyses (see Figure 4).

Summing the rows (or columns) of the between-farm association matrix gave the total farm-level between-farm association frequency. For example, if farm *i *was associated with farm *j *through either sharing the same SH, CC or through being part of an IC, this was represented by 1 in the matrix, or 0 if they were not associated. These industry layers, although considered independent, were combined in the calculation of between-farm association frequency due to lack of knowledge regarding their relative impact on disease transmission potential. Although the strength of contact may vary between these industry layers, their combination provides insight into the range of total associations a farm may have. This has importance for considering the logistics of contact tracing for example, particularly under outbreak circumstances where the importance of different types of contact are not known. No temporally explicit information was available for the inferred between-farm associations, and we note that they may be considered representative of a maximum frequency, since not all associations will be active over any given time period.

## Statistical analyses

### Response variable: between-farm association frequency distribution

**Figure 5:**
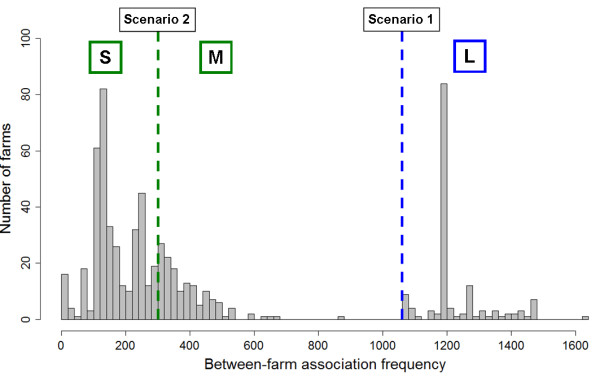
**Distribution of between-farm association frequency and analysis scenarios**. A comparison between large (1079-1623 associations, n = 147 farms) and small/medium (1-879 associations, n = 515 farms) between-farm association frequencies formed scenario 1 analyses, and a comparison between medium (301-897 associations, n = 141 farms) and small (1-299 associations, n = 374 farms) between-farm association frequencies formed scenario 2 analyses. Note: this figure refers to the analysis prior to the removal of records with missing data (i.e. n = 662 farms) and was not qualitatively different following this data reduction.

All farms with a recorded between-farm association frequency ≥1079 were associated with one particularly large SH, resulting in a bimodal frequency distribution (Figure 5). This large SH (black circle, Figure 1) was located in the North of England, but serviced premises throughout GB that represent a range of chicken production types; the majority of their clients were layers (n = 129, 75%), a smaller proportion were broiler breeders (n = 39, 23%) and a small number were broilers (n = 4, 2%), based on data for farms captured by both SH and CC surveys. The between-farm association frequency distribution aggregated farms into two groups; those categorised as 'L' were clearly separate (see Figure 5). This non-standard distribution led to the dichotomisation of the response variable and therefore logistic regression was used.

With the objective of characterising types of PND farms according to their between-farm association frequency, it was considered appropriate to group farms that did not form part of the large SH cluster into two further groups (categorised as small (S) and medium (M), see Figure 5). As there was no epidemiological or practical interpretation of the between-farm association frequency, the choice of cut-off for this dichotomisation of the data was chosen at approximately the mid-point. Whilst this choice was arbitrary, based on an exploratory rationale, it enabled a more direct comparison with scenario 1 analyses than would have been permitted by fitting a more complex continuous distribution. Logistic regression was therefore also used for scenario 2 analyses.

As farms with complete industry contact information were required to determine statistical associations between the farm-level predictors and between-farm association frequency, all farms for which full contact information was not known (i.e. captured by only either SH or CC surveys) were excluded for the purpose of the statistical analyses. This resulted in a reduction in the dataset from 3308 to 662 farm records.

In summary, three between-farm association frequency groups were formed: (i) small (S_af; _1-299 associations, n = 374 farms) (ii) medium (M_af; _301-879 associations, n = 141 farms) and (iii) large (L_af; _1079-1623 associations, n = 147 farms). Based on these categories, two statistical scenarios were formed with different response variables: (i) L_af _versus S_af_/M_af _and (ii) M_af _versus S_af, _referred to as scenarios 1 and 2 respectively (Figure 5). The prevalence of L_af _and M_af _were 22% and 27%, for scenarios 1 and 2 respectively.

### Farm-level predictor variables

A subset of farms (n = 348) with no missing data for the demographic predictor variables were used for the statistical analyses (Figure 4). Following this data reduction, the distribution of farms across the between-farm association categories were as follows: (i) small (S_af; _3-294 associations, n = 183 farms) (ii) medium (M_af; _301-674 associations, n = 87 farms) and (iii) large (L_af; _1079-1623 associations, n = 78 farms). The prevalence of L_af _and M_af _were 22% and 32%, for scenarios 1 and 2 respectively. The possibility that this procedure introduced bias into the statistical analyses was assessed by comparing univariable ORs for the predictor variables, computed both before and after the data exclusion (Tables 1 and 2).

Farm-level predictor variables from the PND were selected for inclusion in the statistical analysis if they were available from the GBPR, and if the proportion of missing observations was not >50% (Table 3). Total farm-level bird count ranged from 2,700 birds - 512,000 birds (median = 77,850 and 48,900 for scenario 1 and 2 data subsets, respectively), and total farm-level poultry house count ranged from 1 - 4 houses (median = 3 for both scenario 1 and 2 analysis data subsets). Numeric (bird count and house count) and management type (indoor and free-range) variables were each grouped into binary small or large and yes or no categories respectively, then re-categorised into their cross-classifications (Table 4). This re-grouping was carried out in order to take account of collinearity (assessed by Pearson's product-moment correlation coefficients ≥ 0.25) without losing information through the exclusion of predictor variables. Furthermore, categorising the numeric variables was useful for interpretation purposes, as the objective was to characterise farms into types based on their demographic profile.

### Data clustering

Due to the complexity of clustering within the PND, multilevel multivariable logistic regression was initially used to control for the data dependency between farms affiliated with integrated companies. However, these models were unstable; three farms with particularly large model residual values had a great influence on scenario 1 model coefficients (ifNY predictor variable was particularly unstable). Despite the instability of the multilevel models, in the subsequent analyses comparing the geographical distribution of between-farm association frequency using the PND with that following extrapolation to the GBPR, they gave qualitatively similar results (not shown). Single-level multivariable logistic regression was therefore considered sufficient.

### Statistical modelling

All statistical analyses were conducted using R v2.92 [57], and models were developed using the *glm *and *glmer *functions for single-level and multilevel models respectively (for *glmer *see lme4 package [58]). All predictors whose coefficients from univariable analyses were associated (p-value ≤0.25) were included in the multivariable models [59]. Model building was carried out manually using a backward reduction method and all potential 2-way interactions were explored between predictors of the most parsimonious model. Model selection was based on the AICc value; a second-order variant of the Akaike Information Criterion [60]. See equations 1 and 2 for the form of the final models corresponding to scenarios 1 and 2 respectively.

(1) $$\begin{array}{c} \text{logit Pr(}{\text{L}}_{\text{af,}i}\text{) }=\beta_{0}+\beta_{1}hbLS_{i}+\beta_{2}hbSL_{i}\\ +\beta_{3}hbLL_{i}+\beta_{4}ifNN_{i}+\beta_{5}ifNY_{i}+\beta_{6}ifYY_{i}\\ +\beta_{7}Integrated_{i}+\beta_{8}East_{i}+\beta_{9}Scotland_{i}\\ +\beta_{10}Wales_{i}+\beta_{11}West_{i}+\beta_{12}ifNN_{i}*Integrated_{i}\\ +\beta_{13}ifNY_{i}*Integrated_{i}+\beta_{14}ifYY_{i}*Integrated_{i} \end{array}$$

(2) $$\begin{array}{c} \text{logit Pr(}{\text{M}}_{\text{af,}i}\text{) }=\beta_{0}+\beta_{1}hbLS_{i}+\beta_{2}hbSL_{i}\\ +\beta_{3}hbLL_{i}+\beta_{4}Integrated_{i}+\beta_{5}East_{i}\\ +\beta_{6}Scotland_{i}+\beta_{7}Wales_{i}+\beta_{8}West_{i} \end{array}$$

The model fit and predictive ability were determined by plotting Studentized residuals and leverage values against the predicted probabilities for each covariate pattern [59], and by obtaining the area under the ROC (Receiver Operator Characteristic) curve for a range of model sensitivities and specificities. The impact on the model coefficients of removing the three most influential data points, as assessed by their Cook's statistic [59], was determined to not have a substantial influence on the model outputs (results not shown).

### Extrapolating network data to the GBPR

Predicted probabilities of a small (*pp_s_*), medium (*pp_m_*) and large (*pp_l_*) between-farm association frequency were obtained for each farm (denoted as *i*) recorded in the GBPR that had no missing data for the corresponding predictor variables (n = 3009). This extrapolation was carried out using a logistic transformation of the linear predictors; coefficients were obtained from the models fitted to the PND, and predictor values were substituted using predictor variable information informed by the GBPR. As large between-farm association frequencies were associated only with a single SH, farms in the GBPR that matched this profile (high *pp*_*l *_value) were considered similar to each other but 'unclassified' with regards to their between-farm association frequency (though for convenience are referred to as L_af_).

### Comparative analysis of geographical variation

For the purpose of comparing the geographical variability between the PND and GBPR, the probability of each GBPR farm having a S_af_, M_af _and L_af _was calculated from the fitted predicted probabilities (see section on 'Extrapolating network data to the GBPR'). These were summarised on a county-average level and compared to the county-average prevalence of observed S_af_, M_af _and L_af _taken directly from the PND (using all the data for which full contact information was known, n = 662) using ArcGIS v.9.2 (ArcView^®^, ESRI, Redlands, CA, USA).

In order to assess at a regional-level the significance of the observed geographical pattern following the extrapolation to the GBPR, 95% confidence intervals were stochastically generated by randomly allocating each farm to a S_af_, M_af _or L_af _group based on their fitted predicted probabilities. This process was repeated for 1000 iterations of randomly allocating farms to a group, enabling the quantification of 2.5% and 97.5% quantiles of the probabilities of S_af_, M_af _and L_af _per region, thus representing the lower and upper bounds of the 95% CIs, respectively (Figures 3a and 3b).

## Authors' contributions

SN designed and conducted all analyses and wrote the manuscript, LM advised on all analyses, PRB and SWJR informed statistical analysis, RRK conceived project and advised on all analyses. All authors read, commented on, and approved the final manuscript.

## References

[B1] Stumpf MP Wiuf C May R Subnets of scale-free networks are not scale-free: Sampling properties of networks Natl Acad Sci USA 2005 102 4221 4224 10.1073/pnas.0501179102 PMC555505 15767579

[B2] Ghani AC Donnelly CA Garnett GP Sampling biases and missing data in explorations of sexual partner networks for the spread of sexually transmitted diseases Stat Med 1998 17 2079 2097 10.1002/(SICI)1097-0258(19980930)17:18<2079::AID-SIM902>3.0.CO;2-H 9789915

[B3] Stumpf MPH Wiuf C Incomplete and noisy network data as a percolation process Journal of the Royal Society Interface 2010 7 1411 1419 10.1098/rsif.2010.0044 PMC2935600 20378609

[B4] Watts CH May RM The influence of concurrent partnerships on the dynamics of HIV/AIDS Math Biosci 1992 108 89 104 10.1016/0025-5564(92)90006-I 1551000

[B5] Tildesley MJ Savill NJ Shaw DJ Deardon R Brooks SP Woolhouse ME Grenfell BT Keeling MJ Optimal reactive vaccination strategies for a foot-and-mouth outbreak in the UK Nature 2006 440 83 86 10.1038/nature04324 16511494

[B6] Vernon MC Keeling MJ Representing the UK's cattle herd as static and dynamic networks Proc Biol Sci 2009 276 469 476 10.1098/rspb.2008.1009 18854300 PMC2592553

[B7] Kiss IZ Green DM Kao RR The network of sheep movements within Great Britain: Network properties and their implications for infectious disease spread J R Soc Interface 2006 3 669 677 10.1098/rsif.2006.0129 16971335 PMC1664651

[B8] Kao RR Green DM Johnson J Kiss IZ Disease dynamics over very different time-scales: foot-and-mouth disease and scrapie on the network of livestock movements in the UK J R Soc Interface 2007 4 907 916 10.1098/rsif.2007.1129 17698478 PMC1975769

[B9] Keeling MJ Woolhouse ME Shaw DJ Matthews L Chase-Topping M Haydon DT Cornell SJ Kappey J Wilesmith J Grenfell BT Dynamics of the 2001 UK foot and mouth epidemic: stochastic dispersal in a heterogeneous landscape Science 2001 294 813 817 10.1126/science.1065973 11679661

[B10] Ferguson NM Donnelly CA Anderson RM Transmission intensity and impact of control policies on the foot and mouth epidemic in Great Britain Nature 2001 413 542 548 10.1038/35097116 11586365

[B11] Kao RR Danon L Green DM Kiss IZ Demographic structure and pathogen dynamics on the network of livestock movements in Great Britain Proc Biol Sci 2006 273 1999 2007 10.1098/rspb.2006.3505 16846906 PMC1635475

[B12] Robinson SE Everett MG Christley RM Recent network evolution increases the potential for large epidemics in the British cattle population J R Soc Interface 2007 4 669 674 10.1098/rsif.2007.0214 17284415 PMC2373390

[B13] Green DM Kao RR Data quality of the Cattle Tracing System in Great Britain The Veterinary Record 2007 161 439 443 10.1136/vr.161.13.439 17906224

[B14] Truscott J Garske T Chis-Ster I Guitian J Pfeiffer D Snow L Wilesmith J Ferguson NM Ghani AC Control of a highly pathogenic H5N1 avian influenza outbreak in the GB poultry flock Proc Biol Sci 2007 274 2287 2295 10.1098/rspb.2007.0542 17644506 PMC2288522

[B15] Sharkey KJ Bowers RG Morgan KL Robinson SE Christley RM Epidemiological consequences of an incursion of highly pathogenic H5N1 avian influenza into the British poultry flock Proc Biol Sci 2008 275 19 28 10.1098/rspb.2007.1100 17956849 PMC2562399

[B16] Dent JE Kao RR Kiss IZ Hyder K Arnold M Contact structures in the poultry industry in Great Britain: exploring transmission routes for a potential avian influenza virus epidemic BMC Vet Res 2008 4 27 10.1186/1746-6148-4-27 18651959 PMC2526082

[B17] Council Directive 2005/94/EC http://archive.defra.gov.uk/foodfarm/farmanimal/diseases/atoz/ai/policy/legislation.htm

[B18] Henzler DJ Kradel DC Davison S Ziegler AF Singletary D DeBok P Castro AE Lu H Eckroade R Swayne D Epidemiology, production losses, and control measures associated with an outbreak of avian influenza subtype H7N2 in Pennsylvania (1996-98) Avian Dis 2003 47 1022 1036 10.1637/0005-2086-47.s3.1022 14575105

[B19] Akey BL Low-pathogenicity H7N2 avian influenza outbreak in Virgnia during 2002 Avian Dis 2003 47 1099 1103 10.1637/0005-2086-47.s3.1099 14575120

[B20] Nishiguchi A Kobayashi S Yamamoto T Ouchi Y Sugizaki T Tsutsui T Risk factors for the introduction of avian influenza virus into commercial layer chicken farms during the outbreaks caused by a low-pathogenic H5N2 virus in Japan in 2005 Zoonoses Public Health 2007 54 337 343 10.1111/j.1863-2378.2007.01074.x 18035971

[B21] Thomas ME Bouma A Ekker HM Fonken AJ Stegeman JA Nielen M Risk factors for the introduction of high pathogenicity Avian Influenza virus into poultry farms during the epidemic in the Netherlands in 2003 Prev Vet Med 2005 69 1 11 10.1016/j.prevetmed.2004.12.001 15899292

[B22] Leibler JH Carone M Silbergeld EK Contribution of company affiliation and social contacts to risk estimates of between-farm transmission of avian influenza PLoS ONE 2010 5 e9888 10.1371/journal.pone.0009888 20360859 PMC2845626

[B23] Capua I Marangon S dalla Pozza M Terregino C Cattoli G Avian influenza in Italy 1997-2001 Avian Dis 2003 47 839 843 10.1637/0005-2086-47.s3.839 14575074

[B24] Halvorson D Karunakaran D Newman JA Avian influenza in caged laying chickens Avian Dis 1980 24 288 294 10.2307/1589789

[B25] Selleck PW Arzey G Kirkland PD Reece RL Gould AR Daniels PW Westbury HA An outbreak of highly pathogenic avian influenza in Australia in 1997 caused by an H7N4 virus Avian Dis 2003 47 806 811 10.1637/0005-2086-47.s3.806 14575068

[B26] Wee SH Park CK Nam HM Kim CH Yoon H Kim SJ Lee ES Lee BY Kim JH Lee JH Kim CS Outbreaks of highly pathogenic avian influenza (H5N1) in the Republic of Korea in 2003/04 Vet Rec 2006 158 341 344 10.1136/vr.158.10.341 16531583

[B27] McQuiston JH Garber LP Porter-Spalding BA Hahn JW Pierson FW Wainwright SH Senne DA Brignole TJ Akey BL Holt TJ Evaluation of risk factors for the spread of low pathogenicity H7N2 avian influenza virus among commercial poultry farms J Am Vet Med Assoc 2005 226 767 772 10.2460/javma.2005.226.767 15776951

[B28] http://archive.defra.gov.uk/foodfarm/farmanimal/diseases/documents/catchersreview.pdf

[B29] Shortridge KF Zhou NN Guan Y Gao P Ito T Kawaoka Y Kodihalli S Krauss S Markwell D Murti KG Characterization of avian H5N1 influenza viruses from poultry in Hong Kong Virology 1998 252 331 342 10.1006/viro.1998.9488 9878612

[B30] Berndtson E Danielsson-Tham ML Engvall A Campylobacter incidence on a chicken farm and the spread of Campylobacter during the slaughter process Int J Food Microbiol 1996 32 35 47 10.1016/0168-1605(96)01102-6 8880326

[B31] Graat EA van der Kooij E Frankena K Henken AM Smeets JF Hekerman MT Quantifying risk factors of coccidiosis in broilers using on-farm data based on a veterinary practice Prev Vet Med 1998 33 297 308 10.1016/S0167-5877(97)00008-1 9500183

[B32] Heyndrickx M Vandekerchove D Herman L Rollier I Grijspeerdt K De Zutter L Routes for salmonella contamination of poultry meat: epidemiological study from hatchery to slaughterhouse Epidemiol Infect 2002 129 253 265 12403101 10.1017/s0950268802007380 PMC2869884

[B33] Ramabu SS Boxall NS Madie P Fenwick SG Some potential sources for transmission of Campylobacter jejuni to broiler chickens Lett Appl Microbiol 2004 39 252 256 10.1111/j.1472-765X.2004.01573.x 15287870

[B34] Cardinale E Tall F Gueye EF Cisse M Salvat G Risk factors for Salmonella enterica subsp. enterica infection in senegalese broiler-chicken flocks Prev Vet Med 2004 63 151 161 10.1016/j.prevetmed.2004.03.002 15158567

[B35] Boender GJ Hagenaars TJ Bouma A Nodelijk G Elbers AR de Jong MC van Boven M Risk maps for the spread of highly pathogenic avian influenza in poultry PLoS Comput Biol 2007 3 e71 10.1371/journal.pcbi.0030071 17447838 PMC1853123

[B36] Dorigatti I PM Rosa R Pugliese A Busani L Modelling the spread of H7N1 avian influenza virus among poultry farms in Italy Epidemics 2010 2 29 35 10.1016/j.epidem.2010.01.002 21352774

[B37] Stumpf MP Wiuf C Incomplete and noisy network data as a percolation process J R Soc Interface 2010 7 1411 1419 10.1098/rsif.2010.0044 20378609 PMC2935600

[B38] Horimoto T Kawaoka Y Influenza: lessons from past pandemics, warnings from current incidents Nat Rev Microbiol 2005 3 591 600 10.1038/nrmicro1208 16064053

[B39] Koopmans M Wilbrink B Conyn M Natrop G van der Nat H Vennema H Meijer A van Steenbergen J Fouchier R Osterhaus A Bosman A Transmission of H7N7 avian influenza A virus to human beings during a large outbreak in commercial poultry farms in the Netherlands Lancet 2004 363 587 593 10.1016/S0140-6736(04)15589-X 14987882

[B40] Martinot A Thomas J Thiermann A Dasgupta N Prevention and control of avian influenza: the need for a paradigm shift in pandemic influenza preparedness Vet Rec 2007 160 343 345 10.1136/vr.160.10.343 17351179

[B41] Horimoto T Kawaoka Y Pandemic threat posed by avian influenza A viruses Clin Microbiol Rev 2001 14 129 149 10.1128/CMR.14.1.129-149.2001 11148006 PMC88966

[B42] Chaves AJ Busquets N Campos N Ramis A Dolz R Rivas R Valle R Abad FX Darji A Majo N Pathogenesis of highly pathogenic avian influenza A virus (H7N1) infection in chickens inoculated with three different doses Avian Pathol 40 163 172 10.1080/03079457.2011.551874 21500036

[B43] Gerritzen MA Lambooij E Stegeman JA Spruijt BM Slaughter of poultry during the epidemic of avian influenza in the Netherlands in 2003 Vet Rec 2006 159 39 42 10.1136/vr.159.2.39 16829597

[B44] Alexander DJ The epidemiology and control of avian influenza and Newcastle disease J Comp Pathol 1995 112 105 126 10.1016/S0021-9975(05)80054-4 7769142

[B45] http://www.defra.gov.uk/food-farm/animals/poultry/

[B46] Evans SJ Sayers AR A longitudinal study of campylobacter infection of broiler flocks in Great Britain Prev Vet Med 2000 46 209 223 10.1016/S0167-5877(00)00143-4 10913805

[B47] van de Giessen AW Bloemberg BP Ritmeester WS Tilburg JJ Epidemiological study on risk factors and risk reducing measures for campylobacter infections in Dutch broiler flocks Epidemiol Infect 1996 117 245 250 10.1017/S0950268800001412 8870621 PMC2271711

[B48] Dent JE Kiss IZ Kao RR Arnold M The potential spread of highly pathogenic avian influenza virus via dynamic contacts between poultry premises in Great Britain. Additional File 1 BMC Vet Res 2011 7 59 10.1186/1746-6148-7-59 21995783 PMC3224601

[B49] Lee KJ Carlin JB Multiple imputation for missing data: fully conditional specification versus multivariate normal imputation Am J Epidemiol 2010 171 624 632 10.1093/aje/kwp425 20106935

[B50] Horton NJ Kleinman KP Much ado about nothing: A comparison of missing data methods and software to fit incomplete data regression models Am Stat 2007 61 79 90 10.1198/000313007X172556 17401454 PMC1839993

[B51] White IR Royston P Imputing missing covariate values for the Cox model Stat Med 2009 28 1982 1998 10.1002/sim.3618 19452569 PMC2998703

[B52] Snow LC Newson SE Musgrove AJ Cranswick PA Crick HQ Wilesmith JW Risk-based surveillance for H5N1 avian influenza virus in wild birds in Great Britain Vet Rec 2007 161 775 781 18065812

[B53] Department for Environment Food and Rural Affairs http://www.defra.gov.uk/food-farm/

[B54] Capua I Marangon S Cancellotti FM The 1999-2000 avian influenza (H7N1) epidemic in Italy Vet Res Commun 2003 27 Suppl 1 123 127 14535379 10.1023/b:verc.0000014128.68876.31

[B55] Halvorson D Karunakaran D Newman JA Avian influenza in caged laying chickens Avian Diseases 1980 24 288 294 10.2307/1589789

[B56] Glass SE Naqi SA Grumbles LC Isolation of avian influenza virus in Texas Avian Dis 1981 25 545 549 10.2307/1589950 6789814

[B57] R Development Core Team R: A language and environment for statistical computing. R Foundation for Statistical Computing, Vienna, Austria http://www.R-project.org

[B58] Package 'lme4'. Linear mixed-effects models using S4 classes http://cran.r-project.org/web/packages/lme4/lme4.pdf

[B59] Hosmer D Lemeshow S Applied Logistic Regression 2000 2 John Wiley & Sons, INC

[B60] Anderson DR Model Based Inference in the Life Sciences. A primer on Evidence 2008 Springer

